# Test-Retest Reliability of the 40 Hz EEG Auditory Steady-State Response

**DOI:** 10.1371/journal.pone.0085748

**Published:** 2014-01-22

**Authors:** Kristina L. McFadden, Sarah E. Steinmetz, Adam M. Carroll, Steven T. Simon, Alissa Wallace, Donald C. Rojas

**Affiliations:** 1 Department of Psychiatry, University of Colorado Anschutz Medical Campus, Aurora, Colorado, United States of America; 2 School of Medicine, University of Colorado Anschutz Medical Campus, Aurora, Colorado, United States of America; University Medical Center Groningen UMCG, Netherlands

## Abstract

Auditory evoked steady-state responses are increasingly being used as a marker of brain function and dysfunction in various neuropsychiatric disorders, but research investigating the test-retest reliability of this response is lacking. The purpose of this study was to assess the consistency of the auditory steady-state response (ASSR) across sessions. Furthermore, the current study aimed to investigate how the reliability of the ASSR is impacted by stimulus parameters and analysis method employed. The consistency of this response across two sessions spaced approximately 1 week apart was measured in nineteen healthy adults using electroencephalography (EEG). The ASSR was entrained by both 40 Hz amplitude-modulated white noise and click train stimuli. Correlations between sessions were assessed with two separate analytical techniques: a) channel-level analysis across the whole-head array and b) signal-space projection from auditory dipoles. Overall, the ASSR was significantly correlated between sessions 1 and 2 (*p*<0.05, multiple comparison corrected), suggesting adequate test-retest reliability of this response. The current study also suggests that measures of inter-trial phase coherence may be more reliable between sessions than measures of evoked power. Results were similar between the two analysis methods, but reliability varied depending on the presented stimulus, with click train stimuli producing more consistent responses than white noise stimuli.

## Introduction

Oscillatory activity in the brain is thought to be an important component of brain function and integration [1], [2]. Neuronal oscillations in the gamma range (∼30–80 Hz) have been implicated in a number of processes, including attention [3]–[5], working memory [6], [7], early sensory processing [8], [9], perceptual binding [10], [11], and language [12]–[15]. Temporal integration of auditory information in the brain can be studied using the auditory steady-state response (ASSR), which was initially investigated by driving the response using clicks presented at 40 Hz [16], [17]. Although steady-state responses are being used increasingly as a marker of brain function, particularly in psychiatric disorders [18]–[21], no studies have investigated the test-retest reliability of this response. Determining the reliability of the ASSR will be essential for the continued use of this response in studies comparing patient and control populations.

Previous studies have found abnormalities in the ASSR in such disorders as autism [20], schizophrenia [18], [21]–[26], and bipolar disorder [19], [27], [28]. Similar abnormalities have also been found in first-degree relatives of patients with these disorders [29]–[31]. The ASSR can be elicited by a variety of stimuli, including clicks presented at high rates or by tone or white noise stimuli amplitude-modulated at the same rates, where 40 Hz driving appears to produce the largest ASSR in adult studies [16], [20], [31]–[38]. Gamma-aminobutyric acid (GABA) is thought to play an important role in the generation of the ASSR [39], [40]. This relationship suggests that ASSR activity may serve as a marker of the efficiency of inhibitory mechanisms in the brain, although most of what is known of this potential is from studies of spontaneous gamma-band responses, not from steady-state responses driven in the gamma-band range [41]–[43]. GABA mechanisms are also thought to be dysfunctional in autism [44]–[46], schizophrenia [39], [47], and bipolar disorder [48], [49]. As such, the ASSR may be an important potential endophenotype (that is, an unseen, heritable phenotype) in these patient populations, and could prove highly useful for assessment during therapeutic development. In addition, this could advance knowledge of the underlying neurophysiology behind these disorders and further genetic and animal studies. Knowledge regarding the test-retest reliability of the ASSR will be important to establish its potential for use as a predictor variable or marker of outcome in clinical trials.

Test-retest reliability has been well documented for various EEG-measured event-related potentials (ERPs) [50]–[54] and for neuronal activity in low frequency bands [55]–[58]. However, relatively few studies have focused on the reliability of auditory responses in the gamma range. While evoked gamma-band activity has demonstrated between-session consistency in response to visual stimuli [43], [59]–[61], little is known regarding reliability in the auditory domain. A preliminary investigation of 6 individuals found amplitude of the peak auditory evoked *transient* gamma-band response to be strongly correlated between sessions in response to white noise stimuli [62]. However, the reliability of the auditory *steady-state* response has not yet been systematically studied. This is an important gap in the literature, since many of the auditory phase-locked gamma-band findings in autism and schizophrenia have emerged from steady-state stimulation approaches.

The purpose of the current study was to investigate between-session reliability of the ASSR as measured by EEG. Based on previous findings, we hypothesized that the ASSR would be significantly correlated between sessions spaced one week apart. While a future study including multiple time points will be important, an initial investigation of the response across one week seemed a reasonable starting point. Further, this time period was chosen with the intent of identifying reliability of the response for an interval of time commonly used in pharmacological trials. Establishing the reliability of this response spaced one week apart without pharmacological intervention will provide important information for future clinical studies utilizing this time period. Previous studies have found the type of stimulus to influence the strength of the ASSR [37], [38], [62]. Stimulus type has also been shown to influence reliability of *transient* responses in the gamma range [62]. As such, the current study assessed differences between the ASSR elicited by both amplitude-modulated white noise and click train stimuli, both of which have previously been found to elicit the ASSR [17], [20], [31], [36], [38]. Furthermore, to evaluate the impact of different analytical techniques on measures of ASSR reliability, two separate analytical methods were compared, one based on sensor-space analyses and the other on source reconstruction. We predicted, based on prior EEG studies of ERP reliability [63], that source reconstruction would improve the signal-to-noise ratio of the measures and result in higher test-retest reliability.

## Methods

### Participants

Nineteen participants (10 male, 9 female, mean age  = 30.1+/−8.8 years, range: 20.3–54.9 years) completed the study. Of these participants, 10.5% identified themselves as African American/Black, 5.3% as Asian, and 84.2% as Caucasian. Ethnic identities were separately ascertained; 21.1% of the sample was Hispanic and the remainder was non-Hispanic. Eligibility criteria required participants to have no personal history of a current or past neurological or Axis I psychiatric disorder, assessed by the SCID Screen Patient Questionnaire-Extended [64]. Participants were recruited via fliers and mass email postings.

### Ethics statement

The human subjects protocol was approved by the Colorado Multiple Institutional Review Board. Written, informed consent was obtained from all participants, consistent with the guidelines of the Declaration of Helsinki.

### Stimuli and paradigm

Participants completed two recording sessions separated by approximately one week (mean  = 10.2, SD = 6.1 days apart, minimum of 5 days between sessions), and completed the same tasks in both sessions. Both were passive listening tasks, in which 200 trials of either white noise or click train stimuli were presented binaurally through foam insert earphones (Neuroscan, Inc., North Carolina, USA) at 75 dB SPL for 500 ms each, with an inter-trial interval of 1000 ms. All participants completed both the white noise and click noise stimuli tasks. White noise stimuli were 500 ms, 40-Hz amplitude-modulated (100 percent depth) white noise. Click stimuli were 40-Hz click trains in which each click was 2 ms in duration delivered every 25 ms for a total of 500 ms. All participants reported having normal hearing. As participant states and attention can impact the ASSR [36], [38], [65], [66], all participants were asked to sit upright and to remain awake with their eyes open during the tasks, with a break given between tasks to maintain alertness. In addition, alpha power was monitored to ensure that participants' alertness did not vary systematically by task or session. Each task condition (white noise and click stimuli) lasted 5 minutes.

### EEG recordings

Continuous EEG data were acquired using a 64-channel electrode cap (EASYCAP GmbH, Herrsching, Germany) with standard 10-10-system electrode placement [67]. Electrodes were placed on the outer canthi of both eyes and on the supra-orbit of the right eye to assess horizontal and vertical eye movements; an additional electrode was placed in the middle of the forehead to serve as the ground. Impedances were below 10 kΩ at all sites. ERP recordings were amplified using Neuroscan SynAmps 2 amplifiers (Neuroscan, Inc., North Carolina, USA), with a passband of .1–200 Hz and digitized at 1000 Hz. Recordings were average-referenced offline. Raw epoch data from this study, along with scripts to read the data, can be downloaded from Figshare (http://dx.doi.org/10.6084/m9.figshare.829584).

### Data preprocessing

Offline, EEG data were preprocessed using Brain Electrical Source Analysis (BESA) 5.3 software (BESA GmbH, Grafelfing, Germany). Data were average-referenced and epochs of 1000 ms were created starting 200 ms prior to stimulus onset and lasting for 800 ms post-stimulus onset. Data were baseline-corrected to the mean of the pre-stimulus period and eye blink artifacts were removed using BESA's spatial filtering routine, which is based on the spatial components method for correcting eye artifacts [68]–[70]. Following eye blink correction, threshold-based artifact rejection was used to remove any epochs with activity greater than 100 µV. Data were then visually inspected and epochs with any additional movement or eye blink artifacts were removed from further analyses. Out of the 200 recorded trials, in the white noise task an average of 180.7 (SD: 23.8) trials were accepted and used for further analyses for session 1, with 184.2 (SD: 17.9) accepted for session 2. For the click train task, an average of 187.1 (SD: 32.9) trials were accepted for session 1, with 178.6 (SD: 20.8) accepted for session 2.

### Statistical analyses

#### Method 1: Sensor-space analysis

Following preprocessing, time-frequency transformation was performed by complex demodulation [71], [72] in BESA, which involves multiplication of the time-domain signal with sines (real) and cosines (imaginary) at each frequency of interest, followed by a Gaussian-shaped finite impulse response (FIR) low-pass filter and calculation of the absolute value. The full width at half maximum was 7.08 Hz and 63 ms, which was the effective resolution of the time-frequency transformation in our study. Within BESA, the time and frequency space was sampled in 2.5 Hz and 20 ms bins for further analyses. Time-frequency representations for both evoked activity, normalized to the pre-stimulus baseline, and inter-trial phase coherence (ITPC) were both derived from BESA [72]–[75] and then imported into Matlab (2009b; MathWorks, Inc., Natick, MA) using FieldTrip routines [76]. ITPC is a measure of event-related phase locking across trials (inter-trial consistency), sometimes referred to as phase-locking factor (PLF), which ranges from 0 (purely non-phase-locked) to 1 (strictly phase-locked) [77], [78]. For both tasks (white noise and click train stimuli), correlation routines in Matlab (using the Statistical Toolbox function, corcoeff.m) were used to determine between-session reliability for evoked activity and ITPC. For each channel (i.e., each of the 63 data channels), each individual time-frequency bin for session 1 was compared to the corresponding time-frequency bin for session 2 across all participants (for both the evoked response and ITPC). This was performed separately for each channel, and a false discovery rate (FDR), as described in Benjamini and Hochberg [79], of *q* = 0.05 was used to correct for multiple comparisons across channels and time-frequency bins. The FDR approach controls for the proportion of false positive findings among findings identified as significant (i.e., a *q* of 0.05 means that no more than 5% of the findings will be false positives). Additionally, the mean correlation coefficients at 40 Hz for each stimulus type (white noise vs. click train) and hemisphere (left vs. right) for absolute power, evoked power, and ITPC, were directly compared for significance using the Fisher r-to-z transformation.

A dependent-samples Student's t-test was run using FieldTrip routines to compare the ASSR (evoked power and ITPC; collapsed across sessions) between the white noise task and the click train task across all channels, using FDR for multiple comparison correction. For visualization purposes, the channel observed to have the highest amplitude at 40 Hz in the 200–500 ms window was identified from the grand averaged response. To assess the signal-to-noise ratio (SNR) for each task (across sessions) at FCz, we first calculated the mean squared coherence (MSC) as the ratio between 40 Hz signal alone (pre-stimulus baseline from −200–0 ms subtracted from the post-stimulus window of 200–500 ms) to 40 Hz signal plus noise (post-stimulus window of 200–500 ms). SNR was then calculated from MSC, as SNR  =  (MSC/[1-MSC])^1/2^ (see [80] for details). Differences in SNR between tasks and hemispheres were assessed using paired t-tests in SPSS version 22 (IBM Corp., Armonk, NY).

#### Method 2: Signal-space projection

**Figure 1:**
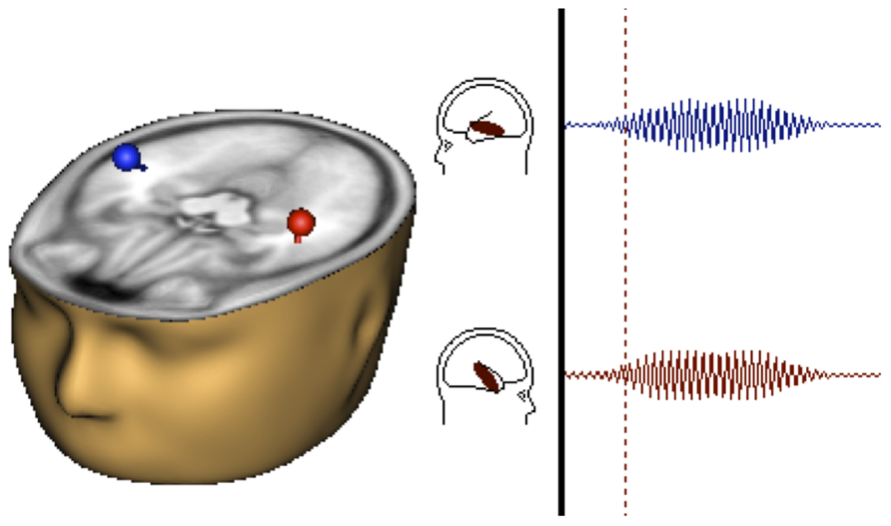
Signal-space projection. Representative diagram of placement of equivalent current dipoles and the resulting signal-space projected averaged waveform resulting from each dipole.

Signal-space projection (also called source-space projection or lead field synthesis [81], [82]) was performed in BESA [73]–[75]. First, a grand average evoked waveform was computed across all participants, using averaged files created from the same preprocessed files used in the whole-head array method. A grand average was created across both tasks (white noise and click stimuli) and both sessions (1 and 2), so that source analysis differences between task and session could not influence the results. Having separate models for each could reduce reliability due to spatial variance between tasks and sessions. As such, there was a single grand average across all participants that included both tasks and sessions. Source analysis was performed by fitting left and right hemisphere equivalent current dipoles to the 40 Hz ASSR in the band-pass filtered (30–50 Hz) grand-averaged response between 200–500 ms (see Figure 1). The left (Talairach coordinates: x = −46.3, y = −18.9, z = −2.6) and right (x = 44.7, y = −13.4, z = 4.9) dipoles were located in primary auditory cortex, and the dipole model residual variance was 8.8%.

This source solution was used to project the raw data for each participant (i.e., the original preprocessed data) into the source domain using a source montage in BESA [83], resulting in a virtual electrode for each participant for left and right hemispheres (i.e., 2 data channels). The projection was done separately for each session (1 and 2) for each task (white noise and click stimuli). Time-frequency transformation was then performed in BESA, for the left and right hemisphere virtual electrodes for each session and task. The same time-frequency transformation parameters as in the sensor-level analysis were used (i.e., complex demodulation: −3 dB power full width at half maximum, resulting in effective time-frequency transformation resolution of 7.08 Hz and 63 ms; time and frequency space within BESA sampled in 2.5 Hz and 20 ms bins). Time-frequency representations for evoked activity and ITPC were then exported to Matlab for between-session correlation analyses. Multiple comparison correction was performed using FDR of *q* = 0.05. Furthermore, the mean correlation coefficients for FCz at 40 Hz for each stimulus type (white noise vs. click train) for absolute power, evoked power, and ITPC, were directly compared for significance using the Fisher r-to-z transformation. These were also compared with correlation coefficients from the sensor-level analysis.

As with the sensor-level analysis, a dependent-samples t-test was run using FieldTrip routines to compare the ASSR (evoked power and ITPC; collapsed across sessions) between the white noise task and the click train task, using FDR for multiple comparison correction. As described above, SNR was calculated from the MSC for each hemisphere for each task, across sessions. Task differences in SNR were assessed using paired t-tests.

#### Fourier analysis

In addition to the time-frequency analyses, Fourier analyses of epochs of the sensor-level and source-level data were conducted. Two time periods were analyzed: 1) between −300 and 0 ms pre-stimulus and 2) between 200 and 500 ms post-stimulus. The pre-stimulus and post-stimulus periods were zero-padded by 1000 ms and Hanning tapered to reduce edge effects. A Fast Fourier Transform (FFT) was used for Fourier analyses. For reliability analyses, the power at 40 Hz in the pre-stimulus and post-stimulus regions, as well as the relative power (post/pre) were statistically analyzed using regression analyses in SPSS. For the sensor-level statistics, power at electrode FCz was used. For the source-level statistics, power was analyzed separately for left and right auditory dipoles. As with the time-frequency analyses, SNR was calculated from the MSC for each task, across sessions. Differences in SNR between tasks were assessed using paired t-tests.

#### Alpha power

Because of the passive nature of the tasks, we also quantified alpha power during the first and last minutes of each 5-minute session. Continuous data for those 60-second periods were segmented into consecutive 1-second Hanning-tapered epochs, upon which an FFT was calculated to derive the power of alpha (8–12 Hz). Only alpha power at electrode Oz was used for this analysis. Differences between the first and last 60 seconds of each session for each task were assessed using paired samples t-tests in SPSS, as were task (white noise vs. click train) and session (session 1 vs. session 2) differences.

## Results

### Method 1: Sensor-space analysis

**Figure 2:**
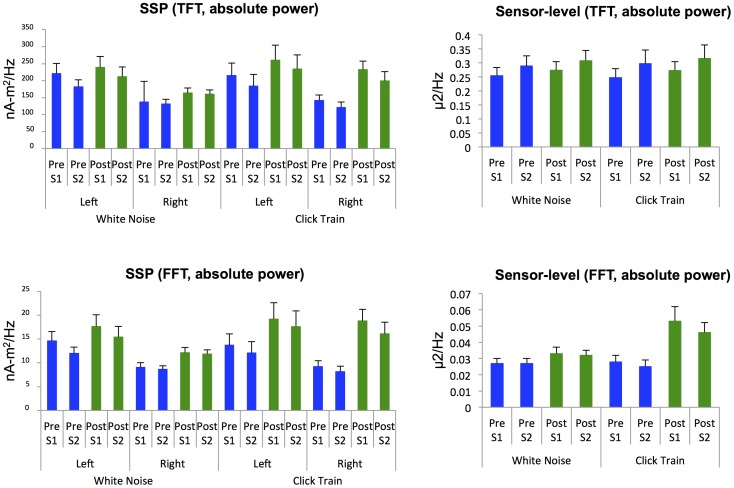
Average absolute power at 40 Absolute power for both the pre-stimulus window (Pre; −200–0 ms for TFT, −300–0 ms for FFT) and post-stimulus window (Post; 200–500 ms), for both session 1 (S1) and session 2 (S2) for both the white noise and click train stimuli. Results are shown for signal-space projected (SSP) and sensor-level (at FCz) data. TFT: time-frequency transformed data; FFT =  Fast Fourier transformed data.

A plot of the grand-averaged response to click train stimuli for each individual channel can be seen in supporting information (Figure S1). Although the click stimuli appeared to evoke a greater ASSR (both evoked and ITPC) compared to the white noise stimuli, differences between the two tasks in either the evoked response or ITPC, assessed across all time-frequency voxels, were not significant after correcting for multiple comparisons. Means for the absolute 40 Hz response in both the pre-stimulus (−200–0 ms) and post-stimulus window at FCz are shown in Figure 2, for both the time-frequency and Fourier transformed data. These data demonstrate significantly increased 40 Hz power to the click train stimuli compared to white noise stimuli in the window of the steady-state response (200–500 ms) for the Fourier analysis (Session 1: *p* = .017, Session 2: *p* = .013). However, this difference was not significant in the time-frequency analysis. The signal-to-noise ratio (SNR) for the ASSR (40 Hz, 200–500 ms) was also calculated across sessions for FCz (see Table 1). Within each task, there were no significant differences in SNR between sessions 1 and 2, in either the time-frequency or Fourier analyses. However, SNR for the click train stimuli was significantly greater than that for the white noise task across both time-frequency and Fourier analyses at FCz.

**Table 1:** Signal to noise ratio (SNR) across sessions for the auditory steady-state response (40 Hz, 200–500 ms).

				Click Train		White Noise		CT vs. WN
	Method	Channel	Measure	Mean	SD	Mean	SD	p
TFT	Sensor	FCz	EP	.33	.12	.29	.10	*
		FCz	ITPC	2.52	.65	1.82	.56	***
	SSP	Left	EP	.44	.21	.30	.14	**
		Right	EP	.75	.34	.47	.18	**
		Left	ITPC	2.05	.68	1.49	.58	**
		Right	ITPC	2.45	.77	1.99	.54	*
FFT	Sensor	FCz	EP	.84	.39	.44	.14	***
	SSP	Left	EP	.60	.25	.42	.19	**
		Right	EP	.93	.43	.61	.22	**

CT =  click train; WN =  white noise; SD =  standard deviation; TFT =  time-frequency transformed data; FFT =  Fast Fourier transformed data; ITPC =  inter-trial phase coherence; SSP =  signal-space projection; EP =  evoked power (absolute). Significant left vs. right hemisphere comparisons: TFT SSP EP (click train***; white noise*); TFT SSP ITPC (click train*; white noise*); FFT SSP EP (click train**; white noise*).

*p<0.05;

**p<0.01;

***p<0.001.

**Figure 3:**
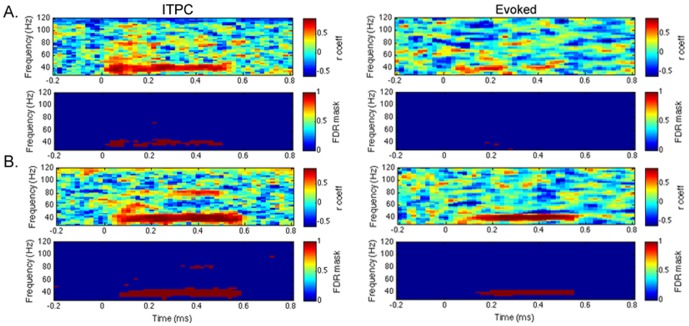
Example of correlation results. Correlation results between sessions 1 and 2 for inter-trial phase coherence (ITPC) and evoked activity for white noise stimuli (A) and click train stimuli (B) for the sensor-level analysis at the channel with the peak 40 Hz auditory steady-state response (FCz). In each plot, the first row shows the correlation coefficient (r coeff) and the second row shows correlations that were significant following multiple comparison correction (FDR, *q* = 0.05; 0/blue  =  not significant, 1/red  =  significant).

The correlation results for the comparison between sessions 1 and 2 for FCz for each task are shown in Figure 3. Plots of significant (*p*<0.05; FDR-corrected for multiple comparisons) correlations between time-frequency bins in session 1 compared to session 2 for each individual channel can be seen in supporting information (Figure S2). Means and ranges of correlation coefficients for the ASSR (40 Hz, 200–500 ms) for FCz are detailed in Table 2 for each task (white noise stimuli and click stimuli) for each measure (evoked power and ITPC). For both tasks, ITPC appeared to be more reliable between sessions than the evoked response. However, comparisons of the mean correlation coefficients at 40 Hz did not find any significant differences between ITPC and evoked responses. Overall, responses to the click stimuli appeared to be more reliable between sessions than those to the white noise stimuli. This can be seen in the figure depicting FCz, in which reliability for both the evoked response and ITPC is more evident for the click stimuli compared to the white noise stimuli. Results from the Fourier analysis are shown in Table 3. As with the time-frequency data, responses to click train stimuli appeared more reliable across sessions at FCz than responses to white noise stimuli. Indeed, the correlation between sessions 1 and 2 was not significant for white noise stimuli, but was significant for click train stimuli. Furthermore, across the whole channel array, a greater number of channels showed statistically significant (*p*<0.05, FDR-corrected) between-session correlations in the range of the ASSR (40 Hz, 200–500 ms) for the click train task (evoked: 29 channels; ITPC: 51 channels) compared to the white noise task (evoked: 7 channels; ITPC: 30 channels). However, when directly comparing the mean correlation coefficients at 40 Hz between click train and white noise stimuli, this comparison was only significant for the normalized evoked response (200–500 ms) at FCZ (see Table 2).

**Table 2:** Correlation values (Pearson's r) between sessions 1 and 2 for the auditory steady-state response (40 Hz): time-frequency analyses.

				Click Train			White Noise			CT vs. WN
Method	Chan	Measure	Time	r	SD	Range	r	SD	Range	p
Sensor	FCz	Absolute EP	Pre	.42	.04	.38–.48	.62	.04	.55–.68	ns
	FCz	Absolute EP	Post	.44	.04	.38–.50	.58	.07	.45–.68	ns
	FCz	Normalized EP	Post	.90	.03	.83–.94	.50	.22	.14–.75	**
	FCz	ITPC	Post	.89	.03	.83–.92	.80	.04	.73–.85	ns
SSP	Left	Absolute EP	Pre	.83	.03	.78–.87	.56	.03	.51–.60	ns
	Right	Absolute EP	Pre	.49	.03	.44–.52	.65	.04	.58–.70	ns
	Left	Absolute EP	Post	.84	.02	.80–.87	.60	.08	.49–.73	ns
	Right	Absolute EP	Post	.73	.05	.63–.80	.69	.05	.60–.76	ns
	Left	Normalized EP	Post	.70	.04	.63–.76	.53	.10	.34–.65	ns
	Right	Normalized EP	Post	.40	.06	.25–.48	.53	.09	.40–.66	ns
	Left	ITPC	Post	.90	.02	.85–.93	.71	.05	.59–.78	ns
	Right	ITPC	Post	.69	.04	.62–.76	.78	.06	.64–.88	ns

SD =  standard deviation; EP =  evoked power; ITPC =  inter-trial phase coherence; SSP =  signal-space projection; Pre =  pre-stimulus window (−200–0 ms); Post  =  post-stimulus window (200–500 ms). Electrode data taken from peak gamma-band electrode (FCz).

**p<0.01.

**Table 3:** Correlation values (Pearson's r) between sessions 1 and 2 for the baseline normalized auditory steady-state response (40 Hz, 200–500 ms): Fourier analyses.

		Click Train		White Noise		CT vs. WN
Method	Measure	r	p	r	p	p
Electrode	FCz Total Power	.73	<.001	.32	.187	ns
SSP	Left Total Power	.42	.074	.53	.020	ns
	Right Total Power	.75	<.001	.61	.006	ns

SD =  standard deviation; SSP =  signal-space projection; CT =  click train; WN =  white noise. Electrode data taken from peak gamma-band electrode (FCz). The correlations reported are for the ratios of post-stimulus/pre-stimulus power at 40 Hz.

As would be anticipated for the click train stimuli, because there is stimulus energy at 40 Hz and its harmonics for this stimulus, a harmonic response at 80 Hz can be seen in both the grand-averaged data and correlations for the click train stimuli, particularly for ITPC. This is also seen, to a lesser extent, in response to the white noise stimuli, suggesting this response is a result of a harmonic to the 40 Hz activity in the brain rather than just to the 40 Hz stimuli, because there is not significant stimulus energy at 80 Hz in the white noise stimulus (above any other frequency). This is an expected effect, as previous studies have found that the brain response to a harmonic can be as strong as that to a stimulus, and may be localized at least partially independently of the fundamental stimulus response generator [36], [84]. The current results suggest that the harmonic responses seen here (at 80 Hz) do show between-session reliability, although perhaps not to the same extent as the response to the fundamental stimulus (40 Hz) (see Figure 3).

### Method 2: Signal-space projection

**Figure 4:**
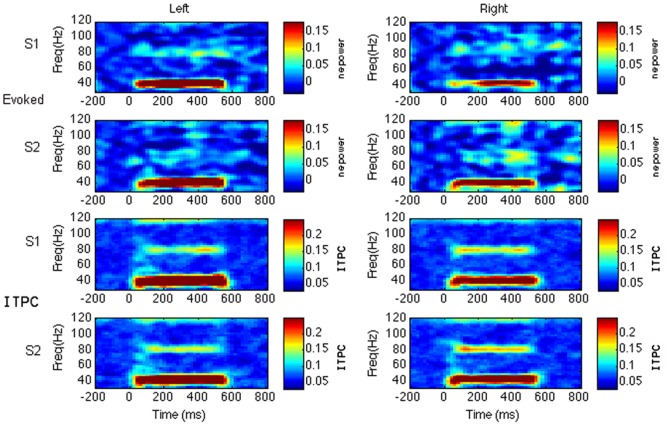
Example of grand averaged data. Time-frequency representations of grand-averaged evoked activity (normalized to baseline) and inter-trial phase coherence (ITPC) in response to click train stimuli for session 1 (S1) and session 2 (S2), for signal-space projected data for the left and right hemisphere. nepower  =  normalized evoked power.

Figure 4 shows time-frequency plots of the grand-averaged evoked power and ITPC for the signal-space projection method for the click train stimuli. The click train stimuli appeared to produce a greater ASSR (evoked power and ITPC), but no significant between-task differences (white noise stimuli vs. click stimuli) survived multiple comparison correction, with the exception of left hemisphere ITPC. In the left hemisphere, the white noise task showed significantly greater ITPC compared to the click train task in the earlier, transient gamma-band response, from 42.5 Hz–52.5 Hz from 40–100 ms (*p*<0.05, FDR-corrected). Conversely, the click train task showed significantly greater ITPC compared to the white noise task in two small areas of the steady-state response, with one time-frequency bin at 32.5 Hz/200 ms and another at 80 Hz/400 ms (*p*<0.05, FDR-corrected). Means for the absolute 40 Hz response in both the pre-stimulus (−200–0 ms) and post-stimulus (200–500 ms) windows are shown in Figure 2, for both the time-frequency and Fourier transformed data. These data demonstrate significantly increased right hemisphere 40 Hz power to the click train stimuli compared to white noise stimuli in the window of the steady-state response (200–500 ms) for session 1 (TFT: *p*<.001; FFT: *p* = .002). The same relationship, albeit only marginally significant, is observed for session 2 (TFT: *p* = .070; FFT: *p* = .057). Additionally, the mean response at 40 Hz for both time-frequency and Fourier analyses was significantly greater in the left hemisphere as compared to the right hemisphere for both the white noise task (session 1, pre-and post-stimulus windows: *p*<.01 for both TFT and FFT; session 2, pre-stimulus window: *p*<.01 for both TFT and FFT; post-stimulus window: *p*<0.05 for TFT) and the click train task (session 1, pre-stimulus window: *p*<.01 for both TFT and FFT; session 2, pre-stimulus window: *p*<0.05 for both TFT and FFT). As with the sensor-space method, a harmonic response can be seen at 80 Hz.

The signal-to-noise ratio (SNR) for the ASSR (40 Hz, 200–500 ms) was calculated for each channel (left and right), across sessions (Table 1). As with the sensor-level analysis, SNR for the click train stimuli was significantly better than that for the white noise task. SNR for the peak 40 Hz channel in the sensor-level analysis (FCz) was similar to that seen for SSP. However, in the right hemisphere, SNR was significantly greater for the SSP approach compared to the sensor-level approach for both click train and white noise stimuli (*p*<.001). In the left hemisphere, SNR was also significantly greater for the SSP compared to sensor-level method for the click train stimuli (*p* = .031), but this was not significant for the white noise stimuli. The same was seen for the Fourier transform analysis, with increased SNR observed for the SSP compared to sensor-level method for right (*p* = .015) and left (*p* = .003) hemisphere for the click train task, and right hemisphere for the white noise task (*p* = .001). SNR for the right hemisphere was greater than that for the left hemisphere, in both time-frequency and Fourier analyses.

The SSP analysis also suggested ITPC to be more reliable between sessions than the evoked response, for both tasks. There were no time-frequency bins surviving multiple comparison correction (*p*<0.05, FDR-corrected) for the evoked response for either task with this method, while ITPC showed significant between-session correlations in the area of the ASSR (40 Hz, 200–500 ms) for both tasks, *p*<0.05, FDR-corrected. As with the sensor-level method, this appears to be more evident for click stimuli compared to white noise stimuli. However, direct comparisons of mean correlation coefficients at 40 Hz from 200–500 ms between (a) ITPC and evoked responses and (b) click train and white noise stimuli, did not find reliability to significantly differ between measure or stimulus type.

### Alpha power

No significant differences in alpha power (8–12 Hz, measured at Oz) were found between the first 60 seconds (M = 1.40, SEM = .38) and the last 60 seconds (M = 1.60, SEM = .51) of session 1 for the white noise task. Similarly, there were no significant differences between the first and last 60 seconds of session 2 for the white noise task (first: M = 1.41, SEM = .59; last: M = 1.25, SEM = .46) or between the first and last 60 seconds of either session for the click train task (session 1, first: M = 1.26, SEM = .46; session 1, last: M = 1.42, SEM = .47; session 2, first: M = 1.18, SEM = .52; session 2, last: M = 1.03, SEM = .45). There were no significant differences in either the first 60 seconds or the last 60 seconds of recording within each session observed between tasks (white noise vs. click), nor were there significant differences in the change in alpha power from session 1 to session 2 between tasks. Furthermore, no significant differences in alpha power during the first and last 60 seconds of recording were observed between sessions (session 1 vs. session 2).

## Discussion

Overall, this study found the auditory steady-state response (ASSR) to be significantly correlated between sessions spaced one week apart, suggesting good test-retest reliability of the response. However, this appears to be more evident for inter-trial phase coherence (ITPC) than for evoked power. The current findings of between-session reliability in the ASSR correspond well with a preliminary study by Jacobson (n = 6) that measured *transient* evoked gamma-band responses to tone stimuli. This study found the amplitude of the early peak 40 Hz response to be significantly correlated between sessions spaced one month apart [62]. The ASSR has been found to be abnormal in a number of patient populations, including autism [20], schizophrenia [18], [21]–[26], and bipolar disorder [19], [27], [28]. However, there is a dearth of information on the reliability of this response. For future applicability to these patient populations, it is important to first establish the reliability of this response and its various measures (e.g., power, ITPC) in a healthy population, as was the aim of the current study.

ITPC was significantly correlated between sessions for both stimuli studied (white noise and click train) and for both analysis methods used (sensor-level and signal-space projection). While ASSR evoked power showed reliability between sessions, this did not survive multiple comparison correction in the signal-space projection analysis, and only a few voxels in select channels survived multiple comparison correction in the sensor-level analysis. Given that ITPC is amplitude-independent and the evoked response is not, making it more susceptible to noise, it is not surprising that ITPC may be more reliable between sessions. However, when directly comparing the mean correlation coefficient at 40 Hz, no significant differences were observed between ITPC and evoked power.

Across both analytical methods, the ASSR to click train stimuli was larger and appeared to be more reliable across sessions compared to the response to white noise stimuli. The reliability of the average 40 Hz normalized evoked response between 200–500 ms (ASSR) was significantly greater for click train stimuli compared to modulated white noise stimuli for the sensor-level analysis. It could be that the more rapid perceptual transition (i.e., on/off) of the clicks is more salient than the sine wave transition in the white noise stimuli, in much the same way that visual contrast is higher in square wave, compared to sine wave gratings [85], [86]. That the strength and reliability of the ASSR was found to differ between stimuli in the current study is not surprising given that previous studies have also found the type of auditory stimulus to influence the size of the response [36]–[38]. Results across the two different analytical methods used (sensor-level and signal-space projection) were very much in agreement. Both methods suggested ITPC may be more reliable than the evoked response, although this is speculative. Furthermore, both methods found click train stimuli to elicit larger and seemingly more reliable steady-state responses compared to white noise stimuli. This was seen in both time-frequency transformed data and Fourier transformed data. This is encouraging for drawing conclusions across multiple studies, as it suggests results using separate analytical methods are comparable.

In the signal-space projection (SSP) analysis, across both tasks, a greater ASSR was seen in the left compared to right hemisphere. It is unlikely that this is due to variation in SNR between the hemispheres, as the right hemisphere demonstrated greater SNR compared to the left hemisphere. Greater SNR in right compared to left hemisphere has also been shown in MEG data [87]. SNR for the click train stimuli was better than that for the white noise task, which could perhaps explain some of the differences in power and reliability between the two tasks. Between the two methods (sensor-level and SSP), the signal-to-noise ratio of the ASSR (40 Hz, 200–500 ms) was similar, but was significantly improved in the right hemisphere for SSP compared to the sensor-level analysis. Of note, in the sensor-level analysis, data from FCz combined activity from left and right hemispheres, whereas in the signal-space projection analysis, data are separately derived for left and right hemispheres using dipoles in auditory cortex. Previous studies have also reported signal-space projection methods to result in a high SNR [36], [74]. One of the challenges of EEG is enhancing the brain signal of interest while reducing the amount of noise in the data. Signal-space projection is a method of spatially filtering the data to separate out the brain activity of interest [74], [88], [89]. Prior research applying dipole analysis techniques to improve auditory ERP reliability also suggests the potential for this approach [63]. SSP also allows a specific area of interest to be chosen as the focus for analyses (i.e., auditory cortex in this case), which could make results more readily interpretable than those from a sensor-level analysis.

While the current study found the ASSR to be robust across sessions spaced one week apart, it remains to be seen if this response will be consistent across longer periods of time. Given that Jacobson's preliminary study [62] found peak *transient* gamma-band amplitude to be reliable across sessions spaced one month apart, it is anticipated that the *steady-state* response will show stability over time periods longer than one week. However, additional studies are needed to confirm this. Because the ASSR is increasingly being investigated as an endophenotype in various disorders, it will also be important to establish the reliability of this response in these patient populations. It could be that reliability of the response is different in various clinical populations compared to healthy controls. However, establishing the reliability of the ASSR in healthy populations is an important first step.

The current study focused on reliability of the ASSR in response to click train and white noise stimuli. While these are commonly used stimuli, particularly in the literature assessing the ASSR as a biomarker in psychiatric disorders [18]–[28], there are other methods of eliciting the ASSR, such as amplitude-modulated sine waves. Future studies should address the reliability of responses elicited by other stimuli than those used in the current study. In addition, a possible limitation of the current study is that the order of stimulus presentation was not counterbalanced. That is, all participants first completed the white noise task, followed by the click train task. The goal of the current study was not to identify order effects, but future studies could include a larger sample size to assess these potential effects. Because of the passive task nature, we used alpha power as a proxy measure of alertness to ascertain if there were systematic differences between sessions or tasks. Alpha power did not differ between the first and last minute of the recording within a session, suggesting that subjects were able to maintain their alertness during tasks. Additionally, no differences in alpha power were observed between sessions (session 1 vs. session 2) or between tasks (click train vs. white noise).

We found that the ASSR is consistent across recording sessions, suggesting that it is reliable over relatively short intervals. However, given that the stability of the response between sessions for evoked activity may not be as robust as for ITPC, and that consistency varied depending on stimulus type, this must be taken into consideration during experimental design. The current findings suggest that click train stimuli elicit a more reliable estimation of the ASSR than do white noise stimuli, perhaps due to the observed higher signal-to-noise ratio in that condition. Finally, we found that signal-space projection and sensor-based analysis methods elicited similar results, although the signal-to-noise ratio was higher for the signal-space projected data, consistent with previous research.

## Supporting Information

Figure S1 **Example of grand average for all channels for sensor-level method.** Time-frequency representations of grand-averaged evoked activity (normalized to baseline) and inter-trial phase coherence (ITPC) in response to click train stimuli for session 1 (S1) and session 2 (S2), for all channels.
(TIF)

Figure S2 **Correlation results for all channels for sensor-level method.** Correlation results between sessions 1 and 2 for inter-trial phase coherence (ITPC) and evoked activity for white noise stimuli (A) and click train stimuli (B) for all channels. Each individual plot shows correlations that are significant following multiple comparison correction (FDR, *q* = 0.05; green = not significant, red  =  significant).
(TIF)
