# Supplementary figures and images for: Test-Retest Reliability of the 40 Hz EEG Auditory Steady-State Response
Source: PLoS One. 2014 Jan 22;9(1):e85748. doi: 10.1371/journal.pone.0085748 (PMC3899078; doi:10.1371/journal.pone.0085748)

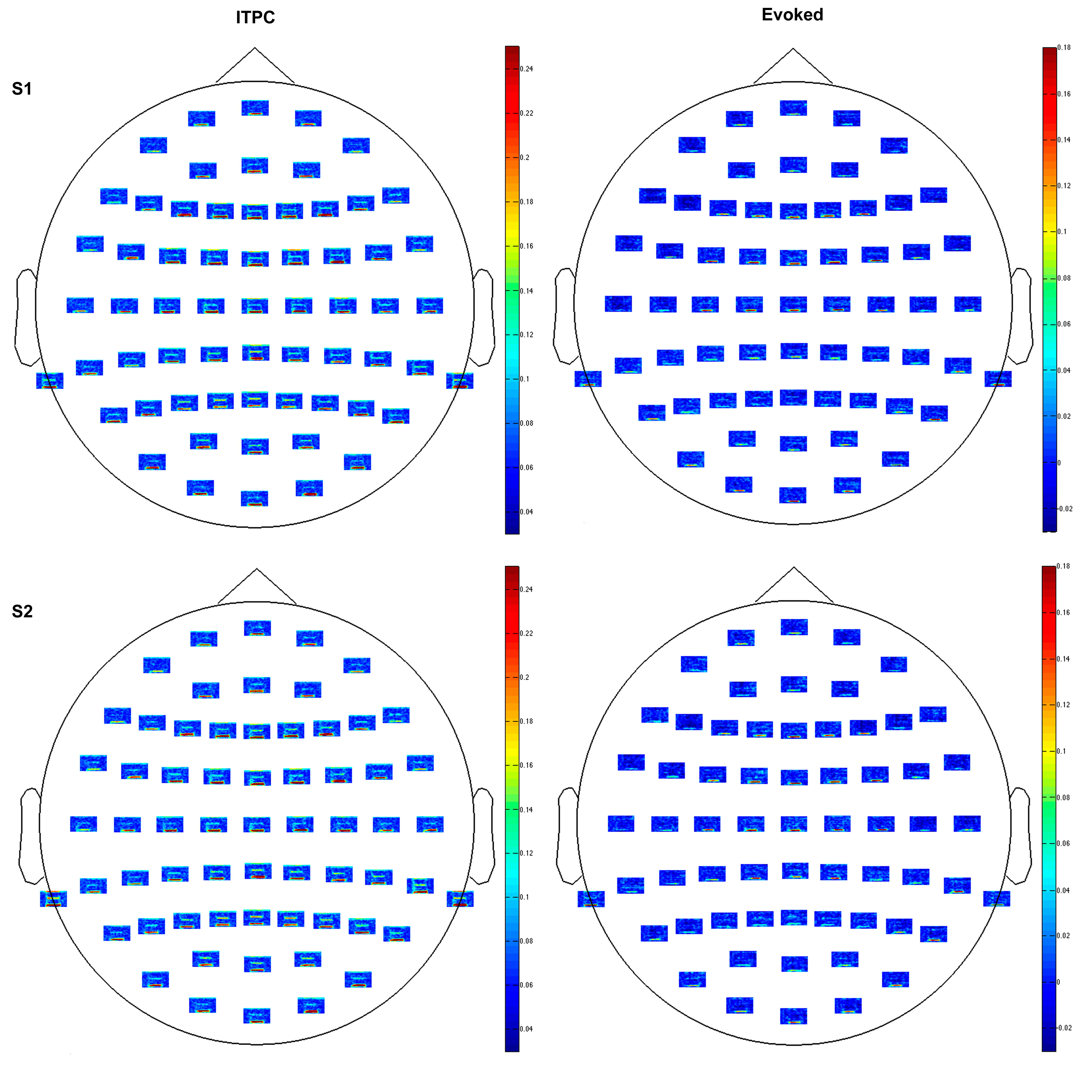

Supplement: Figure S1 — Example of grand average for all channels for sensor-level method. Time-frequency representations of grand-averaged evoked activity (normalized to baseline) and inter-trial phase coherence (ITPC) in response to click train stimuli for session 1 (S1) and session 2 (S2), for all channels. (TIF) [file pone.0085748.s001.tif]

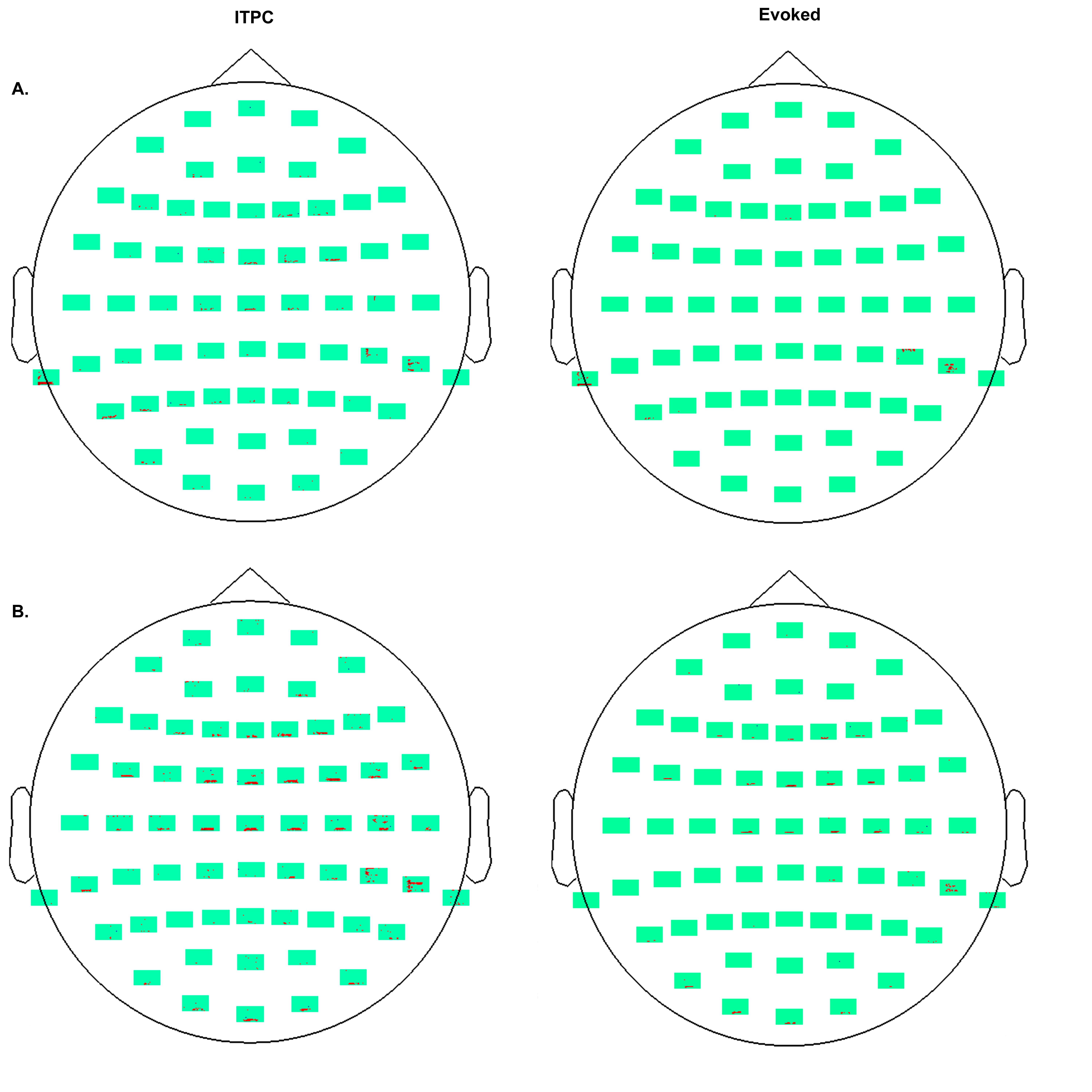

Supplement: Figure S2 — Correlation results for all channels for sensor-level method. Correlation results between sessions 1 and 2 for inter-trial phase coherence (ITPC) and evoked activity for white noise stimuli (A) and click train stimuli (B) for all channels. Each individual plot shows correlations that are significant following multiple comparison correction (FDR, q = 0.05; green = not significant, red = significant). (TIF) [file pone.0085748.s002.tif]
